# IKKepsilon involvement in Tax-mediated activation of INF pathway

**DOI:** 10.1186/1742-4690-8-S1-A137

**Published:** 2011-06-06

**Authors:** Giorgia Cremonese, Francesca Avesani, Erica Diani, Marco Turci, Gianfranco Di Gennaro, Umberto Bertazzoni, Maria G Romanelli

**Affiliations:** 1 Department of Life and Reproduction Sciences, Section of Biology and Genetics, University of Verona, Verona, Italy

## 

HTLV-1 Tax de-regulates several cellular signaling pathways leading to cell transformation by altering gene expression, intracellular protein distribution and cell proliferation. Tax-1 induces persistent activation of several transcriptional factors and signal transduction pathways, including NF-κB and CREB/ATF. It is known that Tax-1 constitutively activates TAK1 (transforming growth factor-β-activated kinase 1) and modifies the interferon (INF) regulatory signals by controlling the expression of INF transcription factors 3 (INF3) and INF4. We have recently reported that HTLV-1 and HTLV-2 Tax proteins interact with TAK1-binding protein 2 (TAB2) of the NF-κB pathway and that both Tax proteins transactivate NF-κB promoters [1]. TAB2 functions as an adaptor protein to recruit TAK1 to TRAF2 (TNF-α receptor-associated factor) in TNF-α signaling pathways.

In the present study we have investigated Tax-1 and Tax-2 role in modifying INF and NF-κB activation through the recruitment of IKKepsilon, an IκB kinase homologue involved in NF-κB and INF3 signaling pathways. By co-immunoprecipitation experiments, we have found that both IKKepsilon and Tax-1, but not Tax-2, are present in protein complexes in transfected cells. IKKepsilon and Tax-1 or Tax-2 role in the activation of INF responsive elements or NF-κB containing promoters have been analyzed after transfecting the protein genes in 293T cells and measuring the effect by luciferase assay. Co-expression of Tax-1 and IKKepsilon resulted in an increased IRF activation mediated by IKKepsilon. Interaction of IKKepsilon with Tax-1 and Tax-2 and their possible effects in the de-regulation of the IRF3 pathways will be discussed.
